# Framework for assessing genetic variation in livestock using demographic, pedigree, and genomic measures

**DOI:** 10.3389/fgene.2026.1792347

**Published:** 2026-04-22

**Authors:** Gábor Mészáros, Ino Curik, Dominique Ouedraogo, Jack Windig, Gregoire Leroy, Yuri Tani Utsunomiya, Pamela Burger, Licia Colli, Chang Xu, Paul Boettcher, Christian Looft, Johann Soelkner

**Affiliations:** 1 Universität für Bodenkultur Wien (BOKU), Vienna, Austria; 2 Department of Animal Science, Faculty of Agriculture, University of Zagreb, Zagreb, Croatia; 3 Institute of Animal Sciences, Hungarian University of Agriculture and Life Sciences, Kaposvár, Hungary; 4 Centre Universitaire de Ziniaré, Joseph KI-ZERBO University, Ouagadougou, Burkina Faso; 5 Wageningen Livestock Research, Animal Breeding and Genomics, Wageningen University and Research, Wageningen, Netherlands; 6 Division of Animal Genetics, Food and Agriculture Organization of the United Nations (FAO), Rome, Italy; 7 Department of Production and Animal Health, School of Veterinary Medicine, São Paulo State University, São Paulo, Brazil; 8 Research Institute of Wildlife Ecology, University of Veterinary Medicine Vienna, Vienna, Austria; 9 Department of Animal Science, Food and Nutrition, Università Cattolica del Sacro Cuore, Piacenza, Italy; 10 Department of Animal Breeding and Husbandry, University of Applied Science Neubrandenburg, Neubrandenburg, Germany

**Keywords:** animal genetic resources (AnGR), effective population size, genetic variation, genomics of diversity, management of genetic resources, population structure

## Abstract

Genetic variation within livestock populations underpins global food security, resilience, and the long-term sustainability of breeding programs. Despite its fundamental role, harmonized approaches for assessing and monitoring genetic variation across data sources remain limited. This review provides an integrated framework for assessing genetic variation in livestock using demographic, pedigree, and genomic data, developed by FAO experts and international collaborators. Demographic indicators offer essential insight into population size, sex ratio, and reproductive structure, while pedigree data allow detailed evaluation of genetic relatedness, inbreeding, and effective population size (*N*_*e*_) over time. Genomic information now provides unprecedented accuracy in characterizing allelic variation, population structure with admixture, and the dynamics of inbreeding and drift. Each data source differs in availability, resolution, and interpretive limits; therefore, complementary use of demographic, pedigree, and genomic measures is recommended for effective monitoring and decision-making. This framework outlines the main properties, applications, and constraints of these approaches and provides guidance on selecting appropriate indicators for monitoring genetic variation within and among livestock populations. Its implementation supports the objectives of the Global Plan of Action for Animal Genetic Resources and the Kunming-Montreal Global Biodiversity Framework, contributing to evidence-based management of livestock diversity worldwide.

## 1 Introduction

The genetic diversity of livestock populations is critical for food security and livelihoods. Genetic diversity allows livestock breeders to modify and adapt their populations in response to changes that occur in the production environment, whether the “environment” is defined as natural factors such as climate, feed availability and endemic diseases, or man-made drivers such as market demand or husbandry practices. The genetic diversity of livestock populations is also a prerequisite for disease resistance, maintaining overall population fitness, prevention of inbreeding problems and genetic improvement of productivity and other economically important traits (Notter, 1999).

Although individual animals are often the private property of farmers, the collective genetic diversity represented across all livestock populations constitutes a public resource that should be managed jointly, for the benefit of society. Consequently, the member countries of the Food and Agriculture Organization of the United Nations (FAO) established a permanent intergovernmental working group under the Commission on Genetic Resources for Food and Agriculture, and adopted the Global Plan of Action for Animal Genetic Resources to guide national and international efforts in the sustainable management of livestock genetic diversity (FAO, 2007).

Genetic variation is influenced by four processes: (i) mutation, (ii) genetic drift, (iii) selection and (iv) migration. Mutations are random changes of the DNA sequence that are the ultimate source of all genetic variation. Theoretically, the effect of beneficial mutations is predicted to be generally small, whereas the frequency of detrimental mutations with a large or even lethal effect tends to remain low. Therefore, mutation has been mostly ignored in animal breeding, at least until the most recent years (Mulder et al., 2019). *Genetic drift* is the random change in allelic frequencies that occurs when a new generation is born. In diploid species, each offspring receives a single allele per locus from each parent, resulting in random sampling of alleles across generations. This stochastic process drives alleles toward fixation, leading to a gradual decline in genetic variation, which proceeds more rapidly as the effective number of breeding individuals decreases. *Selection* is a less-stochastic process that acts on specific loci that are associated with traits influencing an individual’s reproductive success. Like genetic drift, selection tends to decrease genetic variation in the long run, fixing beneficial alleles and eliminating detrimental ones. *Migration* usually increases genetic variation of populations, by introducing alleles that had been rare or absent in the population into which they enter. Given the potential impact that these processes may have on genetic variation within and between populations, it is important to get an overview of the genetic history, in particular the admixture status of the populations of interest.

Various drivers such as population fragmentation or rapid decrease in population size can influence the processes described above, leading to reduction of genetic variation (Leroy et al., 2018). In practice, such a reduction is expected to have two main outcomes: (i) a decrease in fitness, and (ii) a loss of adaptive potential. The emergence of deleterious phenotypes or maladaptive traits in relation to a limited number of variants with large detrimental effects is generally differentiated from the phenomenon of inbreeding depression, although the underlying mechanisms are basically the same (i.e., expression of detrimental alleles). Inbreeding depression is defined as a decrease in fitness or performance of individuals in relation to mating between relatives (Charlesworth and Willis, 2009). The combined effects of all detrimental alleles present in a population constitutes its “genetic load,” which includes the inbreeding load arising from increased homozygosity of recessive harmful alleles. When drift increases the frequency of detrimental alleles faster than selection can eliminate them, the population may go extinct, due to a permanent decrease in fertility, leading to smaller population sizes, more inbreeding, and eventually to a so-called population “meltdown”.

The efficient monitoring of genetic variation is therefore a prerequisite for the sustainable management of genetic resources, particularly animal genetic resources for food and agriculture. The monitoring of trends and risks, indeed, allows the raising of awareness so breeders can act before decrease of genetic variation impacts the fitness of a given breed and puts its very existence at stake. The FAO maintains the Domestic Animal Diversity Information System (DAD-IS; FAO, 2017), a global database that countries can use to monitor the status of livestock populations, by regularly reporting the population sizes of their animal breeds.

The information in DAD-IS is mainly of use for monitoring the amount of genetic variation within livestock species, as indicated by the number of breed populations and the general viability of each population based on its size. However, genetic variation within populations is also important. Recent developments in genomics and biostatistics have provided new opportunities for assessment of genetic variation on the molecular level. On a global and intergovernmental basis, protecting the genetic variation within biological populations has been included as a Target of the recently adopted Kunming-Montreal Global Biodiversity Framework of the Convention on Biological Diversity (CBD, 2022). The member countries of FAO requested further study on the data and parameters that may be used as indicators for monitoring the genetic diversity within livestock breeds.

The objectives of this study were to review the approaches for assessment of genetic variation within livestock populations and to make recommendations for monitoring this variation.

## 2 Monitoring genetic variation within populations

Three sources of information are available to monitor genetic variation within populations. Demographic parameters provide insight into factors driving the loss of genetic variation (e.g., low population size, overuse of popular sires). Pedigree approaches use the knowledge on ancestry to infer the transmission of variants at a locus, allowing the estimation of parameters related to genetic variation such as inbreeding coefficients. Finally, molecular approaches provide direct insight on genomic variation, with information on allele frequencies and homozygosity. These approaches have different merits and limitations, and provide a diversity of measures with different properties, from which a choice has to be made when investigating and monitoring populations.

To be informative and comparable across studies, measures of genetic variation should exhibit well-defined statistical and biological properties. In the first place, they need to be reliable, easy to understand, and accurate to measure. Furthermore, measures should be comparable across species, data sources, populations and time. It is also important that they are easy to determine, easy to interpret, and informative as an early warning indicator in case genetic variation decreases to dangerous levels. As an illustration, measures such as homozygosity or inbreeding may intuitively seem among the best measures of genetic variation. On one hand, both concepts are of utmost importance for the study of genetic variation; homozygosity refers to the occurrence of alleles that are identical (identity by state) at a certain locus for a given individual, while inbreeding is defined here as a measure of shared ancestry in the paternal and maternal lineages (Templeton and Read, 1994). The coefficient of inbreeding (usually abbreviated as *F*) is defined as the probability of homozygosity due to a common ancestor (the probability of identity by descent). Conversely, measures such as homozygosity and coancestry are often challenging to compare among populations, as they can be affected by variation in pedigree depth, marker panels, underlying population structure, and demographic dynamics (Kardos et al., 2015; FAO, 2023). Genetic load may also be quite different between populations, depending on their history of inbreeding and selection, and consequently a high homozygosity or inbreeding level may entail a different risk depending on the population.

Taking the advantages and limitations of these basic measures of genetic variation into context, the effective population size (Ne) has been considered as one of the best metrics of genetic variation and erosion. The Ne is defined as the size of a population following Wright-Fisher assumptions (often referred to as “ideal population”), which quantifies the magnitude of genetic drift and inbreeding in the real-world population under study (Wang et a., 2016). Besides being quite intuitive in terms of interpretation, a big advantage of Ne is that it can be estimated based on various data sources (i.e., demography, pedigree, molecular marker sets) or parameters (e.g., inbreeding, allelic variance). The Ne is also related to the inbreeding within a population through the formula Ne = 1/2ΔF, with ΔF being the change in the rate of inbreeding per generation in the population (Wright, 1931). This is very useful, since the increase in inbreeding approaches the rate at which alleles are eliminated or fixed in a population (genetic drift), while also being directly linked to the increase in risk that genetic defects will be expressed. In other words, Ne and ΔF both measure the loss of genetic diversity occurring in a population. However, Ne as a measure of genetic variation is not without limits. First, as in most populations, genetic variability change is not constant over time; the estimate of Ne can be highly dependent on the period chosen for the analysis. For instance, in many pedigreed dog breeds inbreeding rates have decreased over the past 20 years, as a result of measures taken by breeding organizations to reduce mating of relatives. Consequently, for a given breed, estimates of inbreeding rates considering the most recent generations are low or even negative compared to the estimates considering a longer time period (Windig and Hulsegge, 2021). Also, as the different methods used to estimate Ne consider different assumptions and time periods, uncritical use of different approaches may lead to misleading interpretations of the results.

Overall, understanding the extent of genetic diversity within livestock populations is essential for the sustainable management of animal genetic resources. Three main sources of information can be used to monitor genetic variation: demographic parameters, pedigree-based approaches, and molecular approaches.

### 2.1 Demographic approaches

Demographic measures do not provide genetic information, but give indications on the underlying causes behind the changes in genetic variation present in a population. This information may allow understanding how genetic erosion and inbreeding rates are shaped, and can even provide rough approximates of Ne. However, demographic parameters cannot replace direct information on genetic variation such as from pedigree or molecular information. This section explains how demographic parameters can be used to infer information on genetic variation of the populations.

#### 2.1.1 Population census size

Population size is a direct indicator of the endangerment status related to demographic stochasticity, that is, extinction due to environmental and random catastrophic events (Frankham, 1995a; b; 1996). Census size is also positively correlated to the genetic diversity of a population and its evolutionary potential. In large populations, more genetic variants are expected to be present than in small populations (Gandini et al., 2004). As not all animals reproduce and therefore contribute to future genetic diversity, the number of active breeding females and males is therefore a better indicator than the total number of animals of any age. Demographic trends over time provide additional clues to the fate of a population in terms of genetic variation. High variation of family size, which can be caused by artificial insemination (AI) systems in livestock production, also contributes strongly to the decrease of genetic diversity (Bruford et al., 2015). The reproductive capacity of a species affects the ability of a population to recover. If the habitat of an endangered breed is geographically limited, outbreaks of infectious diseases may put the species at high risk of extinction. The FAO guidelines on *In vivo conservation of animal genetic resources* (FAO, 2013) describe in detail the impact of the major factors influencing risk of extinction and categorizes demographic endangerment status. Categories of risk status of living populations are Critical, Endangered, Vulnerable, Not at risk, Unknown. In its most basic form, assignment to risk status depends on thresholds for census size and reproductive capacity. They can also account for the number of breeding males and females, population trend, and level of pure breeding.

DAD-IS is a tool for monitoring the diversity of animal genetic resources and currently has information on about 8 900 breeds of 37 livestock species in 182 countries, including their risk status. Demographic factors that can be entered are population size (minimum-maximum), trend (increasing, stable, decreasing), breeding males, breeding females, breeding females registered in herdbooks, purebred breeding females, herd size (average), the use of AI, breeding males in AI, males in natural service, and reliability of demographic data (very reliable, reliable, not reliable). The input of minimal and maximal population data is mandatory, all other factors are optional. For a large part of the national breed populations in the system only these two indicators are given, which restrict the information to infer actual genetic diversity of those national breed populations. For instance, data on breeding females and males (used for artificial insemination and natural mating) are completed for only approximately 30 percent of the 15,000 livestock populations in DAD-IS and are rarely in an updated state (accessed 20 September 2025).

#### 2.1.2 Effective population size (Ne)

As previously discussed, the Wright-Fisher idealized population considered for the estimation of Ne is under random mating and no influence of mutation, migration, and selection (Wright, 1931). Some demographic parameters can provide information on the influence of those factors and therefore be used to approximate Ne.

##### 2.1.2.1 Ratio of effective to census population size (Ne/Nc)

Because census population size is often correlated with genetic variation, it can serve as a rough proxy for estimating the effective population size (*N*_*e*_). The equation *N*_*e*_ *= 0.10 × Nc*, where *Nc* denotes the census size, was proposed in the CBD post-2020 Global Biodiversity Framework, as it approximates the mean ratio observed across a wide range of species (Hoban et al., 2020). However, this general relationship may not be directly applicable to livestock populations, as Hall (2016) reported a median *N*_*e*_ */Nc* ratio of 0.03 across more than 500 breeds. Although *N*_*e*_ */Nc* ratio recommendations derived from wildlife studies cannot be directly applied to livestock, the ratio may still provide a valuable preliminary indicator. To enable its meaningful application in livestock, robust reference values should be established through comprehensive research across domestic animal species, considering geographical variation and production system differences.

##### 2.1.2.2 Sex ratio

Data on the actual number of reproducing males and females has been used as a first proxy indicator of Ne (Allendorf et al., 2022). The most commonly used estimator for Ne is based on the unequal sex ratio using the formula:

$$\text{Ne}=4\;\left(\text{Nm }*\text{ Nf}\right)\;/\;\left(\text{Nm}+\text{Nf}\right)$$

where, Nm is the number of reproducing males and Nf is the number of reproducing females.

In livestock species, the number of breeding males is usually much lower than the number of females used for reproduction, so Ne is largely determined by the number of males used.

##### 2.1.2.3 Variance in family size

Unequal contributions of potential parents—or variance in family size—to the genetic composition of the next-generation represent another key factor influencing N_e_. The corresponding relationship can be expressed as follows:

$$N_{e}=\left(8\text{Nm }‐\;4\right)\;/\;\left(\text{Vkm}+\text{Vkf}+4\right)$$

where, Nm is the number of breeding animals and Vkm and Vkf are the variances of the number of offspring of potential male and female parents, respectively, including non-reproducing individuals. Very high Vkm values occur when few sires have a large number of offspring, as is usually the case with AI. Since Vkm is in the denominator of the formula, Ne will be small in such cases. When Vkm = Vkf, the formula is naturally simplified.

##### 2.1.2.4 Variable population size across generations (effect of bottlenecks)

Fluctuations in population size across generations also influence *N*_*e*_. This effect is particularly pronounced when the number of reproducing individuals temporarily declines to a very small size before recovering—a scenario known as a population bottleneck. To account for such fluctuations–this bottleneck effect, the following equation can be applied:

$$\text{Ne}=t\;/\;\left(1/{\text{Ne}}_{1}+1/{\text{Ne}}_{2}+1/{\text{Ne}}_{3}+\ldots +1/{\text{Ne}}_{t}\right)$$

where Ne is the effective population size over a period of t generations, and Ne_1_, Ne_2_, Ne_3_, and Ne_t_ are the effective population sizes of all generations considered in the period of interest. This formula should be used when a genetic bottleneck is suspected. This is the case when the Ne varies greatly (decreases and increases) in successive generations. Note that Ne_1_, Ne_2_, Ne_3_, and Ne_t_ can be estimated using either of the two formulas presented above, taking into account sex ratio or variation of family size. The same formula should be applied for all generations.

It has to be noted that demographic estimates of Ne, and especially the sex-ratio based methods, are merely approximations, and generally yield overestimated values of Ne (Leroy et al., 2013). On the other hand, data required for the sex-ratio Ne estimate (e.g., numbers of males and females reproducing) are usually much easier to obtain than for the family size variation method. For instance, DAD-IS does not include a field for variance of family size. Since 2024, it is possible to upload in DAD-IS effective population size estimates derived from demographic, pedigree or genomic data, resulting in 363 estimates submitted over the last 2 years, covering 176 national breed populations across 15 species in 11 countries. The increasing volume of uploaded data is expected to enhance the monitoring of within-breed genetic variability over time. For details and extensions of the Ne formulas presented here, see Falconer and Mackay (1996), Caballero and Toro, (2020), Allendorf et al. (2022).

#### 2.1.3 Generation interval

The generation interval (L) is defined as the average age of parents at the birth of their offspring (Maignel et al., 1996). It is an important demographic parameter influencing both *N*_*e*_ and the rate of inbreeding over the observation period. In contrast to genetic improvement, where a shorter *L* increases the annual response to selection, a longer *L* is generally desirable for maintaining genetic variation. In most cases, extending the generation interval leads to a higher *N*_*e*_ or a reduced rate of inbreeding over time, as longer intervals reduce the number of generations per unit time and slow the effects of drift and inbreeding. While most demographic estimators of *N*_*e*_ do not explicitly account for the generation interval, pedigree-based indicators typically do so when birth years of individuals are available. Generation intervals vary considerably among livestock species, typically ranging from 2 to 3 years in pigs and sheep to 5–6 years in cattle, but can reach up to 10–11 years in long-lived horse breeds such as the Kladruber and Lipizzaner.

#### 2.1.4 Use of demographic measures for genetic variability monitoring

Despite providing only crude estimates of genetic variability, demographic information is of paramount importance for the management of livestock breeds in general. First, many factors (e.g., epidemics, low number of farms keeping the breed, localized distribution of breeds, low number of animals, high average age of livestock owners) that can contribute to population extinction are more linked to demographic parameters than directly to genetic variability. Second, demographic data can provide valuable information on the causes behind the loss of genetic variation. Despite being probably simpler to collect than pedigree or molecular information, the collection of demographic information is still challenging in many countries. When an animal identification system or regular breed census is not available, cost-effective survey approaches should be implemented to estimate population census size and demographic related measures on a regular basis (FAO, 2004)^1^. The thresholds in *In vivo conservation of animal genetic resources* (FAO, 2013) constitute a solid reference to classify demographic risk status and decide on management or support measures based on this classification.

### 2.2 Pedigree approaches

Over the past two centuries, centralized genealogical registries have become a cornerstone of animal breeding programmes, serving both to unite breeders around defined livestock breed populations and to enable the assessment of kinship relationships among registered individuals (Hill, 2014). Consequently, pedigree data represent a powerful source of information for analysing genetic variation and population structure, as well as for predicting breeding values through the construction of additive relationship matrices. This section will focus on the properties of pedigree approaches and related indicators for the monitoring of genetic variation.

#### 2.2.1 Properties and limits of pedigree approaches

In terms of genetic variability, the knowledge of parent-offspring relationships allows the tracing of gene transmission from generation to generation for an entire population, based on the principle that each parent transfers 50 percent of its DNA to an offspring. It is therefore possible to infer the polymorphism of a given locus, neutral and without mutation, using the probability of either gene identity (a measure of the proportion of genes that are identical within an individual or a population), which allows the estimation of inbreeding and kinship coefficients (additive relationship matrix in a broader contests), or gene origins - a measure of the proportion of genes that have been inherited from a specific individuals/founders or subpopulation (Falconer and Mackay, 1996).

Pedigree approaches have limitations, however. The first limitation is that analyses are restricted to a specific subset of individuals, i.e., those that are currently registered in a herdbook or have been registered in the past (Leroy, 2011). Therefore, an exhaustive breed analysis requires that all individuals are registered in the herdbook, which is not the case with most breeds. Also, investigations are limited to the period during which genealogies have been followed. Depending on the breed, this period may range from a few years to more than a century. As pedigree knowledge may differ over different ancestral lineages due to incomplete registrations, or to the fact that a herdbook may be open for animals whose parents were not registered, heterogenous pedigree knowledge may introduce a bias in pedigree analysis, as relationships between some individuals may not be fully considered. Different metrics are used to assess pedigree depth and gaps. Unknown pedigrees may be inferred to help overcome this bias, while some metrics have been designed to take into account these issues. *Equivalent complete generations* (EqG), i.e., the proportion of ancestors that are known, summed over each generation, constitutes probably the most common metric to assess pedigree completeness (Maignel et al., 1996). Other metrics such as number of complete generations traced or the maximum number of generations traced can also be used to assess contrasts between pedigree depth and pedigree gaps (Boichard et al., 1997; MacCluer et al., 1983; Gutiérrez and Goyache, 2005).

Existence of pedigree errors may constitute another source of bias in the calculation of indicators of genetic variation. However, among livestock breeds, the extent of pedigree errors is typically less than 10 percent (Leroy et al., 2012), whereas Oliehoek and Bijma (2009) observed that errors will have an impact of relevance to conservation decisions only if this percentage exceeds 15 percent.

Finally, it is important to underline that pedigree approaches are based on expectations, thus ignoring stochastic variation, and generally assume neutrality, and therefore cannot be used to reliably infer genetic variation for loci under selection.

#### 2.2.2 Measures of genetic variation

When considering gene identity, two metrics are of utmost importance, namely, the inbreeding (*F*) and kinship, a.k.a. coancestry, (*ϕ)* coefficients. Both indicators measure the probability of two alleles of a given locus to be IBD, either for a single individual, or between two individuals, respectively (Crow and Kimura, 1970). The kinship or coancestry coefficient between two individuals is equal to the expected inbreeding coefficient of a potential offspring of the two individuals. Averaged over a population, both metrics are expected to differ proportionally to an eventual deviation from Hardy-Weinberg equilibrium. The scientific literature recommends focusing on the minimization of average coancestry for monitoring and conservation of genetic variability at the breed level (Baumung and Solkner, 2003; Caballero and Toro, 2000). As IBD is among other things a function of genetic drift that accumulates over generations, pedigree inbreeding and coancestry coefficients tend to increase with pedigree knowledge, which implies that the use of those metrics for comparison of populations needs to consider differences in pedigree completeness among the populations being considered.

Approaches using probabilities of gene origin generally consider the contributions of founders, ancestors or genomes (using their respective effective numbers), which may be of interest for the identification of genetic bottlenecks or to investigate the impact of genetic drift within a given breed (Boichard et al., 1997), those parameters being differentially affected by the extent of pedigree knowledge within the studied population.

Different approaches may be used to investigate population structure with pedigree information. As previously underlined, the simple comparison between average inbreeding and kinship may allow one to identify deviations from Hardy Weinberg equilibrium, which are often linked to the existence of subpopulations or intentional inbreeding (Leroy et al., 2013). Deeper investigation may be considered, using principal component analysis (PCA) (see later) or phylogenetic approaches applied to the “coancestry or additive relationship” matrix (Pirault et al., 2013).

Pedigree based Ne metrics logically use the increase in the rate of IBD over time and generations. They differ on the fact that either inbreeding or kinship are considered and how the period of time or heterogeneity in pedigree completeness are considered. Most common approaches consider the simple increase or linear regression of IBD over a predetermined period (Leroy et al., 2013; Windig and Hulsegge, 2021) or correct individual IBD coefficients by their EqG (Cervantes et al., 2011; Leroy et al., 2020) (Table 1). A variety of software has been developed to analyse pedigree data and compute pedigree metrics, including Ne (Supplementary Annex 1).

**TABLE 1:** Examples of approaches estimating effective population size with pedigree data.

Parameter used	Effective population size formula	IBD rate formula	Time scale considered
Inbreeding rate across generations	$N_{eF}=1/2\underline{\Delta F}$	${∆F}_{t}=\frac{F_{t+1}-F_{t}}{1-F_{t}}$	Adjustable over a given period by linear regression
Individual inbreeding rate		$\Delta F_{i}=1-\sqrt[{{EqG}_{i}-1}]{1-F_{i}}$	Depends on pedigree knowledge
Individual kinship rate	$N_{eC}=1/2\underline{\Delta C}$	$\Delta C_{ij}=1-\sqrt[{{(EqG}_{i}+{EqG}_{j})/2}]{{1-C_{ij}}_{}}$	Depends on pedigree knowledge
Restricted individual kinship rate		$\Delta C_{Rij}=1-\sqrt[{{(EqG}_{\text{Ri}}+{EqG}_{\text{Rj}})/2}]{{1-C_{Rij}}_{}}$	Adjustable on a chosen number of generations

t: Generation number; *F*, *ΔF*: inbreeding and inbreeding rate; *C*_*(R)*_, *ΔC*_*(R)*_: kinship (_*R*_: restricted) and kinship rate; EqG_*(R)*_: Equivalent Complete Generations (_*R*_: restricted).

#### 2.2.3 Considerations on time scale

As previously indicated, inbreeding and coancestry coefficients, as well as Ne measures to a lesser extent, are affected by the pedigree knowledge and time period considered, which may bring bias when monitoring evolution of indicators or comparing different breeds. For instance, in populations with deep pedigree knowledge, changes in breeding practices may lead to relative decreases in inbreeding and kinship, and thus impossibility to estimate Ne. For the sake of comparison, a possibility can be for instance to consider only a restricted number of generations (Leroy et al., 2020). Besides this, given the existence of random fluctuations of inbreeding (and to a lesser extent kinship) levels, that are likely to occur in populations of limited size, it is advised to consider periods larger than two generations and use regression approaches (Pérez-Enciso, 1995) rather than estimating Ne based on two single time points.

#### 2.2.4 Use of pedigree metrics for genetic variability monitoring

According to the *Second Report on the State of the World’s Animal Genetic Resources for Food and Agriculture* (FAO, 2015) the proportions of breeds covered at least partially by pedigree recording was 40 and 51 percent for exotic and locally adapted breeds, respectively, considering the “Big Five” species (cattle, goats, sheep, pigs and chickens). Also, for these five species, between 68 and 91 percent of countries indicated that genetic diversity studies based on pedigree have either not been implemented at all, or only to a small extent (less than 33 percent of breeds). In DAD-IS, herdbooks are reported for fewer than 20 percent of national breed populations. Therefore, genealogical estimates are expected to be potentially available for only a minority of breeds.

For breeds that have generalized genealogical registries, pedigree approaches may however provide useful indicators for breed managers. Pedigree is a common source of data for scientific literature focusing on characterization and monitoring of genetic variability in livestock breeds. For instance, the review of Hall (2016) aggregated from about 90 studies pedigree estimates of Ne from 321 breeds, 31 countries (including 7 countries outside Europe and North America), and five species (cattle, sheep, horses, pigs and goats). Especially for cattle and horses, an increasing number of breeding organizations have integrated pedigree metrics as decision support tools for breeders, allowing for instance assessment of the potential inbreeding of the offspring from a planned mating (e.g., ICBF, 2022). Monitoring systems providing indicators of genetic variability at the breed level in a regular manner are quite rare, however, although some examples exist (see Box 1).

BOX 1 **VARUME: a genetic variability observatory for the ruminants and equid species in France.**

The aim of the project VARUME (Genetic Variability of RUMinants and Equine species) is to set up an observatory of the genetic variability of the French ruminant and equine species, based on pedigree data. It publishes indicators that assess breed genetic variability on a regular basis, by using a common method. In ruminants, the indicators are published on a yearly basis for all cattle and goat breeds with sufficient pedigree information (e.g., mean of at least 2.5 generations of depth) and every 3 years for meat sheep breeds. For instance, in 2021, over 80 individual breed PDF reports were generated (link to resource: https://idele.fr/detail-dossier/varume-resultats-2021). The reports are sent to each breeding organization and are made available publicly on the French Livestock Institute website (IDELE). The main users of the reports are the breeding organizations that are managing breeds with limited genetic variability, typically either endangered breeds or highly selected breeds such as the dairy cattle breeds. The reports allow them to evaluate the efficiency of their management practices to maintain the breeds’ genetic variability. Journalists, researchers and teachers are also using the reports for topics linked to genetic variability. Last but not least, the reports are needed by the French Ministry of Agriculture, as some VARUME indicators can be chosen by the breeding organizations as an indicator of the efficiency of their selection programme. For the equid species, the indicators are calculated on demand by the breeding organizations by using the same methodology. In the equid sector, the breeding organizations generally prefer tools to assess genetic variability at the individual level, such as the calculation of the inbreeding level of a horse and its main contributing ancestor.

While combinations of different estimates may provide interesting insights on historical bottlenecks or substructure within populations, indicators considering the current levels or trends in coancestry probably constitute the most useful metrics for the monitoring of the genetic variability at the breed level. Even with the existence of metrics taking into account heterogeneity in pedigree knowledge and pedigree completeness, the corresponding genetic/time scale should be considered for the interpretation of the results and comparison with other breeds.

### 2.3 Genomic approaches

Unlike demographic and pedigree information, genomic data provide a direct and comprehensive view of the genetic variation present in a population. The field of genomics has undergone remarkable progress in recent years and is poised for continued rapid development. There is a myriad of methods available, not only to determine an animal’s genotype for molecular markers, but also in mathematics and software to infer genetic variation from the molecular data. In this section, common genomic approaches for assessment of genetic variation and their properties are reviewed.

#### 2.3.1 Marker sets and sampling for genetic variability monitoring

The technologies that enable the efficient sequencing of whole genomes have been accessible and affordable for little more than a decade. As such, the widespread use of genome-wide data to characterize genetic diversity is a relatively recent development. The availability of high-quality reference genomes for nearly all livestock species provides many options for assaying diversity, from SNP arrays to various whole genome sequencing (WGS) approaches. A more thorough review of these options can be found in the FAO guide on *Genomic characterization of animal genetic resources* (FAO, 2022a).

There are many types of DNA sequence variants, which can differ in the number of nucleotides, structure, and complexity. Prior decades saw the widespread use of microsatellite markers for the characterization of genetic diversity. They possessed advantages such as being multiallelic and easily assayed *via* a combination of widespread technologies, namely, the polymerase chain reaction (PCR) amplification and capillary electrophoresis (Jarne and Lagoda, 1996). However, genotyping more than a handful of microsatellites per sample can be a very laborious task, thus limiting their utility in representing the diversity and complexity contained in entire livestock genomes. Current technologies are much less laborious and provide a much more detailed insight into genetic variation of livestock for a much lower price. Therefore, the use of microsatellites should be reconsidered, and only done when practical circumstances strongly justify it, such as when a new population is to be added to an existing dataset of microsatellite genotypes (FAO, 2022a). Over the past decade, the use of microsatellites has steadily declined in favor of more advanced technologies such as single nucleotide polymorphisms (SNPs) and whole-genome sequencing (WGS) (Olschewsky and Hinrichs, 2021). SNPs represent the most common form of genetic variation in mammalian genomes. They are typically stable and faithfully transmitted across generations, which makes them highly suitable for tracing genetic variation and inheritance patterns within and between populations. Advances in technology have made it possible to conduct high-throughput genotyping of SNPs. Therefore, they have been widely used to understand the genetic diversity within and between populations (Strucken et al., 2021). Another milestone concerning the genetic variability monitoring was the establishment of whole genome sequencing (WGS), although SNP-arrays remain the dominant tool currently used to characterize the genetic diversity of livestock breeds (Olschewsky and Hinrichs, 2021).

Even when WGS data provide nearly all nucleotides in the genome of each individual, smaller sets of SNPs are often used to represent the genome. Decreasing the resolution of a genomic analysis from the whole genome to a smaller number of variants has the objective to reduce both expenses and complexity. Importantly, the decision on which type and how many variants to select depends largely on the objectives and demands of the intended analysis, in addition to the project budget. Naturally, both SNP arrays and WGS approaches come with a range of advantages and disadvantages. While commercial SNP arrays are available for the most common livestock species, economically marginalized species (e.g., yak, reindeer, guinea fowl) and geographically or genetically isolated populations often lack such resources, either because no arrays have been developed for them or because they are genetically distinct from the breeds used in the design of existing commercial arrays. In the latter cases, low coverage WGS might be the best way to avoid a potential problem called “ascertainment bias”, which is defined as the systematic deviation of population genetic statistics from theoretical expectations (Lachance and Tishkoff, 2013). Many SNPs that are polymorphic in the population on which the SNP array was originally designed may be less variable or even monomorphic in more distant populations. Vice versa, rare alleles existing in the target population most probably will not be captured by the commercial SNP array. This is because the small sample size typically used to develop SNP arrays is more likely to identify loci where both alleles are relatively common than loci with rare alleles. Another important reason to apply whole-genome sequencing (WGS) is to gain insight into the genetic load, which, as discussed earlier, refers to the presence of deleterious (harmful) alleles in selected individuals (Bosse et al., 2019; Huber et al., 2020; Bertorelle et al., 2022). By combining complementary approaches such as Genomic Evolutionary Rate Profiling (GERP) scores and predictions of functional effects, including loss-of-function (LoF) mutations, it is possible to estimate the mutational load—a proxy for genetic load—directly from genomic data (Kutschera et al., 2022). The field of pathogenicity prediction is highly dynamic and continues to evolve rapidly, with ongoing improvements in algorithms, reference datasets, and annotation tools enhancing the accuracy of identifying potentially deleterious variants.

A major question relates to how many samples are needed to ensure that it represents the population and that the estimates are not biased. Different studies (Bhati et al., 2020; Schmidtmann et al., 2021; Mastrangelo et al., 2018) are based on sometimes very different sample numbers (from tens to hundreds of animals). The sample size required depends on the objectives of the study and analyses to be undertaken. Based on these literature results, and because representative sampling cannot always be guaranteed for small populations, for the purpose of evaluating genetic variation it is suggested to sample at least 100 animals (possibly least related and preferentially sex and age balanced) for SNP-genotyping with a 50K array (Manunza et al., 2025). The authors noted that a sample of 50 animals may provide a reasonable approximation and be an acceptable compromise if a sample of 100 animals is impractical. The cost of genotyping 100 animals with a 50K array will be around USD 2 500 for most livestock species in many countries. The cost of sampling is not included in this estimate, however, and will be variable based on the strategy chosen.

For breeds that are genetically distant from the SNP discovery panel, and therefore more susceptible to ascertainment bias, high-density SNP arrays may provide an alternative genotyping strategy, although they are also more expensive.

Concerning the assessment of whether SNP genotyping or WGS should be applied, it must be noted that WGS generally generates more information, but the processing of the data is significantly more difficult compared to the processing of SNP data and costs are therefore much higher. A greater level of technical capacity is required as well. There are numerous well-established software programs available for processing SNP data in diversity studies that can be applied in a user-friendly manner (FAO, 2022a). Nevertheless, if the number of samples is very small, it is recommended to move from SNP analysis to WGS.

#### 2.3.2 Population structure assessment

The structure of a population is important to know because it influences genetic variation, diversity and inbreeding and the interpretation of the results of genomic analysis. Analysing population structure can uncover subpopulations and the patterns of relatedness between them or identify individuals that do not truly belong to the breed being studied (e.g., crossbreds, mislabelled individuals from other populations, *etc.*). Establishing a visual picture of the genetic distribution of animals provides a more complete view of the population structure, with several approaches available such as individual phylogenetic methods, principal component analysis (PCA) or model-based clustering (Olschewsky and Hinrichs, 2021).

PCA is one of the most widely known and used form to visualize population structure. In this approach the variation in all the genomic markers is reduced into a limited number of uncorrelated variables, called “principal components” (Ringnér, 2008). In other words, markers with a strong covariation (either positive or negative) are grouped to form the components. The first two principal components that explain the most variation of the dataset usually indicate groups of individuals with a common genomic make-up. The value of the components can be plotted for each individual, and different groups may become apparent. The relative distance between the groups on this plot shows the similarity between individuals (e.g., Gurgul et al., 2021).

Another widely used approach is to use Bayesian inference to assign individuals on a predetermined number of clusters or groups (Pritchard et al., 2000). Groups are based on having similar genotypes within the groups, and different across groups. At the individual level, genomic origins can be assigned to each cluster as a percentage of its genome. The method is especially useful to determine whether sub-populations as defined (e.g., different breeds) are corroborated by the genomic data.

These different approaches may suffer from inherent biases. For example, Privé et al. (2020) highlighted that PCA-based methods can sometimes capture linkage disequilibrium (LD) structure rather than true population structure, produce shrunk principal components when projecting new datasets into an existing PCA space, or be disproportionately influenced by outliers arising from family structure, population structure, or other sources. Similarly, the model assumptions underlying Bayesian clustering approaches can introduce biases of their own. For instance, such methods may fail to detect subtle population subdivisions, as shown by Lombaert et al. (2018).

Once population structure is determined and subpopulations are defined genetic diversity within and across groups can be analysed using a variety of methods, such as the F-statistics (Wright, 1951), analysis of molecular variance (AMOVA) (Excoffier et al., 1992), inbreeding levels and effective population sizes.

In recent years, advances in deep learning and computational genomics have further transformed the analysis of population structure. New methods based on neural networks, such as POPVAE (Battey et al., 2021) and Neural ADMIXTURE (Mantes et al., 2023), leverage the power of variational autoencoders and neural network architectures to model complex genomic relationships with high accuracy and scalability. These approaches can efficiently process large genomic datasets, capturing subtle population patterns and admixture signals that may be overlooked by conventional statistical methods. As computational resources and algorithmic developments continue to progress, deep learning–based models are expected to play an increasingly important role in uncovering fine-scale population structure and evolutionary relationships across livestock and other species.

##### 2.3.2.1 Genomic methods and tools to estimate effective population size

Genomic data can grasp different signals for the estimation of effective population size (e.g., heterozygote excess, linkage disequilibrium, temporal change in allele frequencies) (Wang, 2005). Currently, the two most prominent methods to calculate Ne based on genomic data are the use of inbreeding (ΔF), and the estimation based on the level of LD (Mészárosová et al., 2021).

The individual autozygosity from data could be estimated using runs of homozygosity (ROH). On the basis on genomic inbreeding levels calculated by using ROH (F_ROH_) in different generations sampled, it is feasible to derive a Ne_ΔF_ indicator. As discussed previously, the change in the inbreeding levels of animals between generations (ΔF) is an indicator of Ne, similarly to the computation of change in F from conventional pedigree data. Accordingly, the same equation Ne = 1/(2*ΔF_ROH_) can be used to compute NeΔF. Established software are available to compute F_ROH_, but no specific software tool is available for the entire process, up to NeΔF. When the F_ROH_ is computed for the entire population, however, one can compute the average ΔF_ROH_ for each generation and follow up with the computation of the corresponding NeΔF, by regressing ΔF_ROH_ on generation number.

Estimation of Ne based on LD is an alternative way to provide valuable information on the population when pedigree information is not available or incomplete. Linkage Disequilibrium describes a non-random association of alleles at different loci and is a function of the recombination rate between these loci on a physical map (Evans and Cardon, 2005). It can result from various demographic changes, like admixture and genetic drift, (Wright, 1931), or the “hitchhiking effect” during selection (Charlesworth et al., 1997; Maynard and Haigh, 2007). According to Sved (1971), LD between unlinked loci can arise from genetic drift from an isolated population with random mating. Hence, Ne can be estimated by the known relationship of and variance in LD among various loci, which can be calculated by allele frequency, which may reflect the genetic drift over generations. While there are many measures of LD, the most used one is pairwise values. This LD-based estimation method has been widely used for conservation programmes and the study of the evolution of local populations (Waples and Do, 2010; Santiago et al., 2020; Santiago et al., 2025). With the rapid development of genotyping methods, software for prediction based solely on genomic data has also become available, with various software available for estimating historical changes from genotype data (Supplementary Annex 1). While the genetic methods to estimate contemporary Ne are generally accurate, model biases, such as genotyping errors, non-random sampling, age or spatial structure might distort the results. An adjustment of experimental design might be also needed based on the studied species (Waples, 2025).

#### 2.3.3 Molecular monitoring of breeds

According to the Second Report on the State of the World’s Animal Genetic Resources for Food and Agriculture (FAO, 2015), among the main species (cattle, chicken, goat, pig, sheep), between 73 and 84 percent of countries indicated that within-breed molecular genetic diversity studies - had either not been implemented at all, or only to a low extent (less than 33 percent of breeds). This result suggests that obstacles likely exist for general monitoring of molecular monitoring of breeds worldwide. Molecular approaches have reportedly been used at a similar level to pedigree data for the computation of Ne in scientific literature, and in the review of Hall (2016), about 30 studies had estimated NeLD for 203 breeds, 30 countries (including 9 countries outside Europe and North America), and 5 species (cattle, sheep, horses, pigs and goats).

Despite the accuracy that molecular measures provide, to our knowledge there is no monitoring system in place using this source of information to follow up genetic variation of livestock in a regular manner. This gap between availability of tools and their application (Bruford et al., 2015; Leroy et al., 2018) are likely to be related to lack of technical, organizational, financial capacity at field level. This continuous monitoring is recommended, as the genetic variation of livestock breeds is expected to change over time. Depending on the result of such monitoring, breeders and breeding organizations could take action if necessary to maintain genetic variation and minimize increases in inbreeding.

## 3 Assesment of genetic variation using demographic, pedigree and genomic

Based on the information presented in the sections above, it is important to describe the extent to which the different sources of information display different properties that affect their utility for population monitoring (Table 2). For instance, demographic parameters provide unique and indispensable information for monitoring extinction risk related to demographic stochasticity and reveal the existence of factors that can drive changes in genetic variation. In the case of exhaustive genealogical registration, demographic parameters of importance can be extracted from the pedigree file. Genomic information, if obtained with appropriate marker sets, sampling and approaches, is likely to provide the most accurate picture of genetic variation. However, if the purpose is population monitoring, genomic approaches should always be used in complementarity to demographic or pedigree analyses.

**TABLE 2:** Properties and challenges related to the use of demographic, pedigree and molecular approaches for population monitoring.

Data source	Main properties	Challenges	
		Data collection	Applicability of results
Demographic information	Provides insight on demographic stochasticity and underlying causes behind the changes in genetic variation	Requires collection of data through breed censuses, surveys or animal identification systems	Estimates of genetic variation are basic interpolations which often underestimate loss of genetic variation
Pedigree information	Provides inferences on the genetic variation of selectively neutral loci and assuming no mutation, based on knowledge of parent-offspring relationships	Requires registration of pedigree information to be as complete as possible	Results do not consider mutation, Mendelian sampling and selection, and are prone to bias related to incomplete or incorrect pedigree
Genomic information	Yields information directly on genomic variation, but provide no direct information on demographic stochasticity	Requires accurate sampling in terms of individuals and markers	Choice of appropriate parameters used for analyses requires skill, and may yield inaccurate results if parameters are incorrect

The priority assigned to a given approach depends largely on the situation of the breed under study. For instance, for a population with a very large number of registered animals with complete and correct pedigree information over several generations, demographic and genealogical measures will sufficiently describe the situation of the breed in terms of genetic variation, although complementary DNA-based analyses can provide additional details about other aspects of the population. For another population with only a small number of animals, and for which the pedigree relationships are largely unknown, basic characterization of demographic parameters complemented with genomic characterization would be necessary to obtain accurate assessment of the diversity and genetic structure of the population. Genomic studies are also generally the only approach available when the goal of a given analysis is the study of across-breed diversity, where demographic data and pedigrees would reveal no genetic relationship between groups.

The use of measures for comparison across time periods and between different breeds and subsequent decision-making needs to be carefully considered. For instance, it has been shown that demographic predictors of Ne tend to provide upwardly biased estimates relative to genealogical or molecular approaches, since the demographic approaches do not consider several factors of importance (Leroy et al., 2013; Hall, 2016). Therefore, the same rules of decision (e.g., Ne thresholds to prompt a given action) should not be applied indiscriminately for demographic *versus* pedigree/molecular estimates. Therefore, if measures of inbreeding or Ne from different approaches are to be used as global indicators of genetic diversity and/or collected in a breed monitoring database like DAD-IS, complementary information (i.e., estimation approach used, citation of a reference providing more information on details such as the subpopulation sampled) should be provided along with the data for the estimates.

## 4 Conclusion and recommendations

This review presents an integrated framework for assessing and monitoring genetic variation in livestock populations by combining demographic, pedigree, and genomic data. Effective monitoring of genetic diversity is essential for the sustainable management of animal genetic resources, as it provides the basis for informed decisions on breeding, conservation, and risk assessment. While demographic and pedigree information have long formed the basis for evaluating population structure, inbreeding, and risk status, the increasing accessibility of molecular tools now provides unprecedented opportunities to quantify genetic variation with greater accuracy and comparability across breeds and regions, while also enabling the estimation of admixture and introgression. The complementary use of these data sources enables better understanding of genetic erosion processes, population dynamics, and the effectiveness of management practices over time. Based on the evidence and expert consensus, the authors recommend that genetic variation within livestock populations be regularly assessed using at least one indicator of effective population size (*N*_*e*_), and that such monitoring be conducted at intervals corresponding to the generation interval of the species. Estimates of *N*_*e*_ should be integrated into the Domestic Animal Diversity Information System (DAD-IS), in line with the Kunming–Montreal Global Biodiversity Framework, which recognizes *N*_*e*_ as a key indicator of within-population genetic diversity. Molecular tools, particularly medium-density SNP arrays and whole-genome sequencing, provide substantially higher precision than demographic or pedigree approaches, especially in cases where the quality of pedigree information is limited. It is recommended to sample 100 animals per breed, including both sexes and a range of age classes, to representatively capture variation across multiple generations. Whenever available, genomic information should be used in conjunction with demographic and pedigree data, to strengthen temporal continuity and interpretability of results. An even higher emphasis should be placed on genomic data in case of large, and intensively selected populations. For classification of breeds by risk status, the thresholds and procedures defined in the *FAO Guidelines on In vivo Conservation of Animal Genetic Resource* (FAO, 2013) remain the appropriate reference framework. Integration of *N*_*e*_ and related measures into national monitoring systems, breeding programs, and conservation plans will strengthen the capacity of countries and breeders to safeguard livestock genetic diversity, support adaptation to changing environments, and contribute to the implementation of the Global Plan of Action for Animal Genetic Resources.

## Footnotes

1
FAO (2022b). Collection and estimation of population size data for risk classification in DAD-IS = A sampling methodology. Other documents.
