# Supplementary material for: Framework for assessing genetic variation in livestock using demographic, pedigree, and genomic measures
Source: Front Genet. 2026 Apr 22;17:1792347. doi: 10.3389/fgene.2026.1792347 (PMC13143417; doi:10.3389/fgene.2026.1792347)
Supplement: Supplementary file 1 [file Supplementaryfile1.docx]

Annex 1

**Examples of pedigree and genomic analysis software**

**PEDIGREE ANALYSIS SOFTWARE**

ENDOG

Gutiérrez, J. P., & Goyache, F. (2005). A note on ENDOG: a computer program for analysing pedigree information. *Journal of Animal Breeding and genetics*, *122*(3), 172-176.

<https://webs.ucm.es/info/prodanim/html/JP_Web.htm>

Pedig

Boichard, D. (2002). Pedig: a fortran package for pedigree analysis suited for large populations. In *7th world congress on genetics applied to livestock production* (p. Inconnu).

<https://www6.jouy.inrae.fr/gabi_eng/Support-Expertise/Software/Pedig>

PMx

Lacy, R. C., Ballou, J. D., & Pollak, J. P. (2012). PMx: software package for demographic and genetic analysis and management of pedigreed populations. *Methods in Ecology and Evolution*, *3*(2), 433-437.

<https://scti.tools/pmx/>

PyPedal

Cole, J. (2012). PyPedal, an open source software package for pedigree analysis. *Eur. Assoc. Anim. Prod. Proc*, *18*, 239.

<http://pypedal.sourceforge.net/>

Grain v2.0

Baumung R, Farkas J, Boichard D, Mészáros G, Sölkner J, Curik I. GRAIN: a computer program to calculate ancestral and partial inbreeding coefficients using gene dropping approach. J Anim Breed Genet. 2015;132:100–8.

Doekes HP, Curik I, Nagy I, Farkas J, Kövér GY, Windig JJ. Revised calculation of Kalinowski’s ancestral and new inbreeding coefficients. Diversity. 2020;12:155.

https :// boku.ac.at/nas/nuwi/software or <https://angen.agr.hr/hr/group/37/Grain+2-2>

Retriever

Windig, J. J., & Hulsegge, I. (2021). Retriever and pointer: Software to evaluate inbreeding and genetic management in captive populations. *Animals*, *11*(5), 1332.

<https://genebankdata.cgn.wur.nl/software/Retriever/Retriever.html>

**GENOMIC ANALYSIS SOFTWARE**

PLINK

Chang, C. C., Chow, C. C., Tellier, L. C., Vattikuti, S., Purcell, S. M., & Lee, J. J. (2015). Second-generation PLINK: rising to the challenge of larger and richer datasets. *Gigascience*, *4*(1), s13742-015.

<https://www.cog-genomics.org/plink/>

cgaTOH

Zhang L, Orloff MS, Reber S, Li S, Zhao Y, *et al.,* (2013) cgaTOH: Extended Approach for Identifying Tracts of Homozygosity. PLOS ONE

<http://www.cs.kent.edu/~zhao/TOH/>

detectRuns

Biscarini, F., Cozzi, P., Gaspa, G., & Marras, G. (2018). detectRUNS: Detect runs of homozygosity and runs of heterozygosity in diploid genomes. CRAN (The Comprehensive R Archive Network).

<https://orca.cardiff.ac.uk/id/eprint/108906/>

RZooRoH

Druet T, Gautier M. A model-based approach to characterize individual inbreeding at both global and local genomic scales. Mol Ecol. 2017;20.

<https://cran.r-project.org/web/packages/RZooRoH/vignettes/zooroh-vignette.pdf>

GONE

Santiago, E., Novo, I., Pardiñas, A. F., Saura, M., Wang, J., and Caballero, A. (2020). Recent demographic history inferred by high-resolution analysis of linkage disequilibrium. Mol. Biol. Evol. 37 (12), 3642–3653. doi:10.1093/ molbev/msaa169

https://github.com/esrud/GONE

GONE2

Santiago, E., Köpke, C. & Caballero, A. Accounting for population structure and data quality in demographic inference with linkage disequilibrium methods. Nat Commun 16, 6054 (2025). <https://doi.org/10.1038/s41467-025-61378-w>

https://github.com/esrud/GONE2

NeEstimator v2.X

Do, C., Waples, R. S., Peel, D.,Macbeth, G.M., Tillett, B. J., and Ovenden, J. R. (2014). NeEstimator v2: Re-implementation of software for the estimation of contemporary effective population size (Ne) from genetic data. Mol. Ecol. Resour. 14 (1), 209–214. doi:10.1111/1755-0998.12157

https://github.com/bunop/NeEstimator2.X
